# NETosis and NADPH oxidase: at the intersection of host defense, inflammation, and injury

**DOI:** 10.3389/fimmu.2013.00045

**Published:** 2013-03-01

**Authors:** Nikolaos G. Almyroudis, Melissa J. Grimm, Bruce A. Davidson, Marc Röhm, Constantin F. Urban, Brahm H. Segal

**Affiliations:** ^1^ Division of Infectious Diseases, Department of Medicine, University at Buffalo School of MedicineBuffalo, NY, USA; ^2^Department of Medicine, Roswell Park Cancer InstituteBuffalo, NY, USA; ^3^Department of Anesthesiology, University at Buffalo School of MedicineBuffalo, NY, USA; ^4^Department of Pathology and Anatomical Sciences, University at Buffalo School of MedicineBuffalo, NY, USA; ^5^Veterans Administration of Western New York Healthcare SystemBuffalo, NY, USA; ^6^Laboratory for Molecular Infection Medicine Sweden, Department of Clinical Microbiology, Umeå UniversityUmeå, Sweden; ^7^Department of Immunology, Roswell Park Cancer InstituteBuffalo, NY, USA

**Keywords:** NETs, NADPH oxidase, neutrophils, inflammation, injury

## Abstract

Neutrophils are armed with both oxidant-dependent and -independent pathways for killing pathogens. Activation of the phagocyte nicotinamide adenine dinucleotide phosphate (NADPH) oxidase constitutes an emergency response to infectious threat and results in the generation of antimicrobial reactive oxidants. In addition, NADPH oxidase activation in neutrophils is linked to activation of granular proteases and generation of neutrophil extracellular traps (NETs). NETosis involves the release of nuclear and granular components that can target extracellular pathogens. NETosis is activated during microbial threat and in certain conditions mimicking sepsis, and can result in both augmented host defense and inflammatory injury. In contrast, apoptosis, the physiological form of neutrophil death, not only leads to non-inflammatory cell death but also contributes to alleviate inflammation. Although there are significant gaps in knowledge regarding the specific contribution of NETs to host defense, we speculate that the coordinated activation of NADPH oxidase and NETosis maximizes microbial killing. Work in engineered mice and limited patient experience point to varying susceptibility of bacterial and fungal pathogens to NADPH oxidase versus NET constituents. Since reactive oxidants and NET constituents can injure host tissue, it is important that these pathways be tightly regulated. Recent work supports a role for NETosis in both acute lung injury and in autoimmunity. Knowledge gained about mechanisms that modulate NETosis may lead to novel therapeutic approaches to limit inflammation-associated injury.

## INTRODUCTION

Neutrophils are armed with a broad repertoire of tools for killing pathogens. Activation of the phagocyte nicotinamide adenine dinucleotide phosphate (NADPH) oxidase constitutes an emergency response to infectious threat and results in the generation of antimicrobial reactive oxidant intermediates (ROIs). In addition to oxidant-dependent host defense, neutrophils harbor proteases, antimicrobial peptides, lactoferrin, and other antimicrobial constituents that damage and kill microbes. Activation of NADPH oxidase in neutrophils is linked to activation of intracellular granular proteases and to generation of neutrophil extracellular traps (NETs). NETs are composed of an extracellular network of chromatin bound to granular and specific cytoplasmic proteins that can target extracellular pathogens.

NETosis can be induced *in vitro* by conditions that lead to robust NADPH oxidase activation [e.g., stimulation with phorbol myristate acetate (PMA)], and is triggered *in vivo* during states of emergency, such as infection and conditions mimicking sepsis, such as transfusion-associated acute lung injury (Caudrillier et al., 2012; Yipp et al., 2012). In this setting, NETosis in dead or dying neutrophils may amplify microbial killing. Whereas neutrophil apoptosis leads to non-inflammatory physiological cell death, NETosis results in the extracellular release of proteases and other injurious neutrophil constituents that can exacerbate inflammatory injury (Narasaraju et al., 2011; Caudrillier et al., 2012; Thomas et al., 2012).

We review the link between NETosis and NADPH oxidase activation and their effects on host defense and modulation of inflammation and injury. A greater understanding of these pathways may lead to novel therapeutic approaches to limit inflammation-associated injury.

## HOW NEUTROPHILS DIE

One of the most important aspects of regulation of acute inflammation relates to its termination. Neutrophils recruited to sites of microbial invasion or tissue injury are activated by microbial products (e.g., endotoxin and formylated peptides), damage-associated molecular patterns (DAMPs; Zhang et al., 2010), and cytokines and chemokines within the inflammatory milieu. While this acute inflammatory response is critical for host defense, subsequent termination of neutrophilic inflammation and transition to inflammatory responses that mediate tissue repair (e.g., M2 or alternative macrophage polarization; Sica and Mantovani, 2012) are necessary to limit tissue injury.

Neutrophil homeostasis involves production and death of an extraordinarily large population of cells. The lifespan of circulating neutrophils has been estimated to be <1 day; however, a recent *in vivo* labeling study showed that the average estimated circulating human neutrophil lifespan was considerably longer (5.4 days; Pillay et al., 2010). During infection and other major stressors, granulopoiesis and circulating neutrophil counts can increase dramatically; these conditions also modulate neutrophil survival and death as well as the mode of death.

Apoptosis is the default mode of neutrophil death. Apoptosis is stimulated by a number of factors [e.g., members of the tumor necrosis factor (TNF) cytokine family], and is regulated by specific caspases and members of the Bcl-2 family of proteins (Croker et al., 2011; Geering et al., 2011). Factors that can delay neutrophil apoptosis include lipopolysaccharide (LPS), granulocyte colony-stimulating factor (G-CSF), granulocyte-macrophage colony-stimulating factor (GM-CSF), and proinflammatory cytokines (Coxon et al., 1999; Klein et al., 2001; Garlichs et al., 2004; Akagi et al., 2008; Jun et al., 2011). Neutrophils are likely committed to apoptotic death by their constitutive co-expression of cell-surface Fas and Fas ligand, an autocrine mechanism that is suppressed by proinflammatory cytokines (Liles et al., 1996). In mice, phagocytosis of apoptotic neutrophils by macrophages reduces IL-23 production by these cells and downstream IL-17A production by T cells, thereby limiting granulopoiesis (Stark et al., 2005).

NETosis and necrosis of neutrophils are induced by different stimuli and have morphologically distinct features. Following toxin-induced necrosis, nuclear lobes in neutrophils lose their hypersegmented structure, but the nuclear envelope and granules remain intact (Fuchs et al., 2007). Nuclear decondensation and breakdown of the nuclear and granular membranes are unique features of NETosis (Fuchs et al., 2007). NETs are demonstrated by immunofluorescence showing mixing of nuclear (e.g., DNA and histones) and granular constituents [e.g., neutrophil elastase (NE)] on the extracellular surface of neutrophils (Brinkmann et al., 2010).

How neutrophils die likely affects their clearance and cross-signaling to monocytes/macrophages. At sites of inflammation, neutrophils undergo spontaneous apoptosis (Savill et al., 1989). In addition, neutrophil apoptosis can be induced by macrophages releasing death receptor ligands, such as TNF-α and Fas ligand (Brown and Savill, 1999; Yamashita et al., 1999; Renshaw et al., 2000). Macrophages recognize and ingest apoptotic neutrophils (Savill et al., 1989). Phosphatidylserine products are externalized by neutrophils early during apoptosis and stimulate phagocytosis of neutrophils by macrophages (efferocytosis), thus promoting resolution of inflammation (Frasch et al., 2011). In contrast, NETotic neutrophils display phosphatidylserine only after plasma membrane rupture (Fuchs et al., 2007). In addition, release of primary neutrophil granular proteins can recruit circulating monocytes to the site of inflammation, stimulate macrophages to produce cytokines, and enhance the ability of macrophages to phagocytose bacteria (Soehnlein et al., 2008a, 2009a,b).

## GENERATION OF NETs

Neutrophil extracellular trap generation was first described by Brinkmann et al. (2004), who showed that neutrophils release granule proteins and chromatin that co-mingle in extracellular filaments that bind to and kill bacteria and degrade virulence factors. NETosis progresses through stages that are distinct from apoptosis and necrosis. The nuclei of neutrophils transition from hypersegmented lobes to a round, decondensed morphology. Peptidylarginine deiminase 4 (PAD4) converts histone tail arginine residues to citrulline, leading to loss of positive charge and chromatin decondensation (Neeli et al., 2009; Wang et al., 2009; Li et al., 2010; Farley et al., 2012). Later, the nuclear envelope and the granule membranes break down, allowing mixing of these components that are subsequently released as extracellular structures (Fuchs et al., 2007). Whereas NETosis has been considered to be an event following neutrophil death and breakdown of membranes, it is also possible for viable neutrophils to produce NETs (Yousefi et al., 2009; Yipp et al., 2012). Yipp et al. (2012) showed that during experimental infection, anuclear neutrophils with intact membranes formed NETs and were capable of migration and phagocytosis.

NETosis can be triggered by exposure of neutrophils to PMA, IL-8, LPS, interferon-gamma (IFN-γ ), bacteria and fungi and their products, and complement-mediated opsonization (Brinkmann et al., 2004; Martinelli et al., 2004; Fuchs et al., 2007; Yamada et al., 2011; Saitoh et al., 2012; Yipp et al., 2012). Activated platelets can induce NETosis in neutrophils during bacterial sepsis, thereby capturing microbes and promoting their clearance (Clark et al., 2007; Phillipson and Kubes, 2011; McDonald et al., 2012). The proportion of activated neutrophils undergoing NETosis, the rapidity of NET generation, and the dependence on specific signaling pathways vary based on the stimulus (Hakkim et al., 2011). The Raf–MEK–ERK pathway is an upstream activator of NADPH oxidase in neutrophils and is involved in NETosis (Hakkim et al., 2011). Raf–MEK–ERK also upregulates expression of Mcl-1, an anti-apoptotic protein, suggesting that Raf–MEK–ERK may inhibit apoptosis to allow for NETosis (Hakkim et al., 2011). Several other signaling pathways can modulate NETosis. Inhibition of autophagy prevents chromatin decondensation in neutrophils and abrogates NETosis (Remijsen et al., 2011). Mammalian target of rapamycin (mTOR) mediates LPS-stimulated NET formation by post-transcriptional control of expression of hypoxia-inducible factor-1 alpha (HIF-1α; McInturff et al., 2012). IFN-γ produced by neutrophils can stimulate NETosis through an autocrine/paracrine process (Yamada et al., 2011).

In addition, specific neutrophil components that are NET constituents are also required for NET generation. NE is required for NET generation in pneumonia in mice (Papayannopoulos et al., 2010). In this model, NE traffics from primary granules to the nucleus where, together with myeloperoxidase (MPO), it degrades histones and promotes chromatin decondensation. MPO is also located in neutrophil primary granules and converts hydrogen peroxide to hypohalous acid, which has potent antimicrobial properties. MPO deficiency in humans leads to failure to generate NETs following stimulation with PMA or *Candida* (Metzler et al., 2011).

Neutrophil extracellular traps are also regulated by inhibiting pathways. SerpinB1 is an inhibitor of the neutrophil serine proteases. SerpinB1-deficient mice develop increased inflammation, tissue injury, and mortality following bacterial and viral infection (Benarafa et al., 2007; Gong et al., 2011). SerpinB1 restricted NETosis through a pathway that involves translocation of SerpinB1 from the cytoplasm to the nucleus early during NETosis (Farley et al., 2012). Curiously, recombinant SerpinB1 also inhibited NETosis through mechanisms that are unclear (Farley et al., 2012). MUNC13-4, a member of the MUNC13 family of proteins, is involved in exocytosis of lytic granules in cytotoxic T lymphocytes, and its deficiency leads to familial hemophagocytic lymphohistiocytosis (Feldmann et al., 2003). In neutrophils, MUNC13-4 mediates ROI generation, exocytosis of primary granules, and phagolysosomal maturation, but also inhibits NETosis (Monfregola et al., 2012). Finally, serum endonuclease DNase1 promotes degradation of NETs (Hakkim et al., 2010). Pathways that inhibit NETosis may limit neutrophil-mediated injury.

## RELATIONSHIP BETWEEN NADPH OXIDASE AND NETosis

The phagocyte NADPH oxidase is comprised of a membrane-bound cytochrome consisting of gp91^*phox*^ (phagocyte oxidase) and p22^*phox*^. Upon activation, the cytoplasmic subunits, p47^*phox*^, p67^*phox*^, and p40^*phox*^ and rac translocate to the cytochrome. NADPH is oxidized to NADP^+^, and electrons are transported down a reducing potential gradient that terminates when oxygen accepts an electron and is converted to superoxide anion. Neutrophil NADPH oxidase activation occurs in response to microbes and to stimuli that can mimic infectious threat, such as formylated peptides, opsonized particles, integrin-dependent adhesion (Mocsai et al., 2002; Graham et al., 2007), and to activation of specific pathogen recognition receptors, such as dectin-1, which recognizes fungal cell wall-associated beta-glucans (Gantner et al., 2003; Goodridge et al., 2011).

Chronic granulomatous disease (CGD), an inherited disorder of NADPH oxidase, is characterized by recurrent life-threatening bacterial and fungal infections. Patients with only residual NADPH oxidase activity in neutrophils have a better prognosis than those with completely absent oxidase function (Kuhns et al., 2010). CGD is also associated with severe inflammatory complications, such as Crohn’s-like inflammatory bowel disease (Marciano et al., 2004; Segal et al., 2011).

Activation of NADPH oxidase in neutrophils is linked to the release of cationic proteins from an anionic proteoglycan matrix within primary granules (Reeves et al., 2002). In this model, the released neutrophil serine proteases become activated and can target phagocytized microbes. NADPH oxidase can also stimulate NETosis. PMA-stimulated NET generation requires NADPH oxidase, while MIP-2 induces NETosis independently of NADPH oxidase (Farley et al., 2012). In pneumococcal lung infection, NETosis of lung neutrophils was reduced, but not eliminated, in NADPH oxidase-deficient mice (Yamada et al., 2011). Neutrophils from CGD patients are defective in NETosis, and gene therapy results in restored NETosis in NADPH oxidase-competent neutrophils *in vitro* (Fuchs et al., 2007; Bianchi et al., 2009).

Neutrophil NADPH oxidase can also modulate apoptosis. NADPH oxidase stimulates phagocytosis-induced apoptosis (Coxon et al., 1996). TNF-α and Fas ligand can both induce apoptosis in neutrophils, but through distinct signaling pathways; NADPH oxidase was required for TNF-α-stimulated, but not Fas ligand-stimulated, apoptosis (Geering et al., 2011). Accelerating neutrophil death and clearance are likely to be important modes by which NADPH oxidase limits acute inflammation. Potentially, the intensity or kinetics of ROI generation may modulate the balance between apoptosis versus NETosis.

## NADPH OXIDASE AND NETs IN HOST DEFENSE

NADPH oxidase can potentially target pathogens through a multi-step process: direct antimicrobial effect of ROIs; intracellular activation of proteases that can target phagocytized pathogens; and generation of NETs that can attack extracellular pathogens. NETs can mediate host defense by trapping microbes thereby limiting their spread and by exposing them to high concentrations of several antimicrobial products. NET constituents can target different pathogens. For example, NE can degrade certain virulence factors of pathogens (Belaaouaj et al., 2000; Weinrauch et al., 2002). Calprotectin is a NET constituent that mediates nutritional immunity by sequestering divalent metal ions and targets *Candida* and *Aspergillus* species (Urban et al., 2009; Bianchi et al., 2011). However, the contribution of NET generation to host defense *in vivo* is difficult to determine because there are no genetic defects that selectively disable NETosis while leaving all other immune pathways intact. Therefore, it remains elusive whether the major role of NETs is to trap versus directly kill pathogens *in vivo*. In addition, the biological activity of individual cellular components released into NETs is undetermined. With these gaps in knowledge, the host defense contribution of NETosis versus that achieved by intracellular killing by intact neutrophils remains unsettled (Nauseef, 2012).

Specific pathogens may target NETs or exploit NET products to enhance bacterial invasion. For example, nuclease expression by *Staphylococcus aureus* degrades NETs and augments pathogen virulence *in vivo* (Berends et al., 2010). In addition, bacteria may exploit NET products to enhance microbial virulence. *Shigella flexneri* binds to cationic granular proteins expressed in NETs, which enhances bacterial adherence to and invasion of epithelial cells (Eilers et al., 2010). Paradoxically, excessive NETosis may impair host defense *in vivo*. SerpinB1-deficient mice had increased lung neutrophil NET generation but defective bacterial clearance compared to wild type mice in *Pseudomonas aeruginosa* pneumonia (Farley et al., 2012). While SerpinB1 may be required for host defense independently of its role in NETosis, these results raise the potential for injury caused by excessive NETosis abrogating antibacterial killing.

Neutrophil elastase and MPO are located in neutrophil primary granules, are constituents of NETs, and are required for NET generation in response to specific stimuli (described above). Therefore, deficiencies in these pathways can provide some insight into the role of NET constituents in host defense. The neutrophil serine proteases, NE, cathepsin-G (CG), and proteinase 3 (PR3), are activated by lysosomal cysteine protease cathepsin C/dipeptidyl peptidase I (DPPI; Pham et al., 2004). Papillon–Lefèvre syndrome is a rare autosomal recessive disease resulting from loss-of-function mutations in the *DPPI* gene locus that is characterized by palmoplantar hyperkeratosis, periodontitis leading to loss of teeth, and severe bacterial infections, including liver abscesses (Almuneef et al., 2003; Pham et al., 2004). MPO-deficient mice have defective candidal killing *in vivo* (Brennan et al., 2001) and neutrophils from MPO-deficient patients are impaired in the ability to limit growth of extracellular *Candida albicans* (Metzler et al., 2011). However, MPO deficiency in humans is usually asymptomatic, although severe candidal infections have been observed in patients with co-existing diabetes (Cech et al., 1979a,b).

NADPH oxidase deficiency leads to a more severe phenotype than Papillon–Lefèvre or MPO deficiency. For example, patients with CGD are at high risk for invasive aspergillosis and specific bacterial pathogens (e.g., *Burkholderia cepacia*, *Serratia marcescens*, and *Nocardia* species; Winkelstein et al., 2000; van den Berg et al., 2009). Consistent with these clinical observations, CGD mice were highly susceptible to infection by *Aspergillus* and by *B. cepacia*, while NE^-/-^ × CG^-/-^ mice and DPPI-deficient mice were resistant (Vethanayagam et al., 2011).

Additional studies point to specific host defense functions of neutrophil proteases that are distinct from NADPH oxidase. Proteases in human neutrophils target *Streptococcus pneumoniae* (Standish and Weiser, 2009) and protease-deficient mice have increased susceptibility to pneumococcal pneumonia (Hahn et al., 2011). In contrast, neutrophil NADPH oxidase has variable effects on pneumococcal strains *in vitro*; CGD neutrophils have normal killing of pneumococci that produce peroxide, but defective killing of peroxide-deficient mutant strains (Pitt and Bernheimer, 1974; Shohet et al., 1974), suggesting that pathogen-derived reactive oxidants can complement the killing defect in CGD neutrophils.

Together, these observations in the clinic and in mouse models support NADPH oxidase, neutrophil serine proteases, and MPO having distinct functions in host defense. Interventions that deplete NETs *in vivo* (e.g., histone blocking antibody and DNase1; Caudrillier et al., 2012) may be useful to delineate specific host defense functions of NETs.

## NADPH OXIDASE AND NETs HAVE DISTINCT EFFECTS ON INFLAMMATION AND INJURY

The generation of NETs can be a double-edged sword. On the one hand, they may promote pathogen killing. However, the same pathways that control microbial infection can also cause injury through a number of mechanisms. NET constituents can damage epithelial and endothelial cells, which can exacerbate inflammation-induced organ injury (Saffarzadeh et al., 2012). The interaction between NETs, platelets, and coagulation further illustrate the concept of the dual host defense and injurious potential of NETs. Platelet-derived defensins have intrinsic antimicrobial activities and can stimulate NETosis (Kraemer et al., 2011). NET constituents (NE, CG, and histones) can activate platelets and promote coagulation leading to intravascular thrombus growth that restrict tissue bacterial invasion (Fuchs et al., 2010, 2011; Massberg et al., 2010); however, these same mechanisms may promote an excessive coagulopathy and thrombosis resulting in endothelial cell injury and organ damage. Recent studies have shown that NETosis can drive transfusion-related acute lung injury and that depleting NETs was protective (Caudrillier et al., 2012; Thomas et al., 2012).

NETosis has also been implicated in the pathogenesis of autoimmune disorders, such as systemic lupus. Autoimmunity may be driven by a combination of factors related to aberrant NETosis, including direct damage to tissue, release of autoantigens that stimulate immune complexes, complement activation, and IFN-α release by plasmacytoid dendritic cells (Guiducci et al., 2010; Hakkim et al., 2010; Meyer-Hoffert and Wiedow, 2010; Garcia-Romo et al., 2011; Lande et al., 2011; Villanueva et al., 2011; Leffler et al., 2012). A subset of patients with systemic lupus have reduced ability to degrade NETs due to impaired serum DNase1 activity, and have a higher incidence of lupus nephritis (Hakkim et al., 2010).

While NETosis has, so far, been shown to enhance neutrophil-mediated injury, the role of NADPH oxidase is more complex and likely involves interplay of injurious and protective pathways. NET constituents such as neutrophil proteases generally lead to augmented inflammation and tissue injury (Adkison et al., 2002; Hu and Pham, 2005; Raptis et al., 2005; Akk et al., 2008; Soehnlein et al., 2008b). However, studies in NADPH oxidase-deficient mice point to a more complex interaction between NADPH oxidase and acute lung injury that is context-dependent. NADPH oxidase worsened the severity of acute lung injury following influenza virus challenge in mice (Imai et al., 2008). While wild type and NADPH oxidase-deficient mice had similar levels of lung injury following LPS challenge (Sato et al., 2002), NADPH oxidase-deficient mice had greater lung neutrophil sequestration, but less lung injury compared to wild type mice in *E. coli* sepsis (Gao et al., 2002). In contrast, NADPH oxidase was protective in acid aspiration-induced lung injury. NADPH oxidase-deficient mice had increased airway neutrophilic inflammation and acute lung injury (measured as albumin leak), but less injury per recovered neutrophil, compared to wild type mice (Segal et al., 2007; Davidson et al., 2013). In addition, NADPH oxidase was required for optimal activation of Nrf2, an ROI-inducible transcriptional factor that stimulates cytoprotective and anti-inflammatory responses (Davidson et al., 2013). Thus, NADPH oxidase likely has a dual effect on inflammation-induced lung injury. While the immediate effects of NADPH oxidase activation leads to ROI generation and possibly NETosis that are predicted to be injurious, NADPH oxidase can also protect against injury by limiting neutrophilic inflammation and by inducing cytoprotective pathways, such as Nrf2.

## CONCLUSION

In response to infectious threat, neutrophils go to war. NADPH oxidase is rapidly activated by specific microbial stimuli, leading to ROI generation. NADPH oxidase can activate neutrophil granular proteases within the phagolysosome, thereby targeting phagocytized pathogens. In addition, NADPH oxidase stimulates NETosis, a process that targets extracellular pathogens. While important for host defense, NETs are also implicated in the pathogenesis of a number of diseases, including acute organ injury and autoimmunity. Understanding mechanisms by which NADPH oxidase and its regulated pathways modulate inflammation and injury may identify novel therapeutic approaches.

There are several gaps in knowledge regarding NETosis. Although NETosis is induced by infection or conditions mimicking infectious threat, we do not have a good understanding of the molecular mechanisms that drive NETosis. NADPH oxidase can stimulate NETosis in certain settings and apoptosis in others; it is unclear how these dual effects are mediated. It is also unclear which pathways stimulate NETosis after neutrophil death and breakdown of membranes versus NETosis in living neutrophils. Additional questions relate to the relative contributions of NETosis versus intracellular killing of pathogens to host defense and whether the major function of NETs is to prevent spread of versus killing of pathogens.

Future translational areas of research involve the application of NETotic products as prognostic biomarkers for inflammatory disorders (e.g., sepsis, autoimmunity). For example, in patients with sepsis, the degree of NETosis may correlate with a better outcome due to enhanced bacterial clearance or a worse outcome reflecting inflammation-induced organ injury. Assays of NETotic markers (e.g., MPO–DNA complexes, NE) from cell-free fluid (e.g., plasma) may provide a quantitative measure of NETosis from archived human samples that could be correlated with clinical outcome in a variety of diseases. This knowledge may pave the way to therapeutic approaches to target NETs, such as use of histone neutralizing antibody or DNase1.

## Conflict of Interest Statement

The authors declare that the research was conducted in the absence of any commercial or financial relationships that could be construed as a potential conflict of interest.
